# Pessaries in multiple pregnancy as a prevention of preterm birth: the ProTwin Trial

**DOI:** 10.1186/1471-2393-9-44

**Published:** 2009-09-17

**Authors:** Maud A Hegeman, Dick J Bekedam, Kitty WM Bloemenkamp, Anneke Kwee, Dimitri NM Papatsonis, Joris AM van der Post, Arianne C Lim, Hubertina CJ Scheepers, Christine Willekes, Johannes J Duvekot, Marc Spaanderman, Martina Porath, Jim van Eyck, Monique C Haak, Marielle G van Pampus, Hein W Bruinse, Ben Willem J Mol

**Affiliations:** 1 Department of Obstetrics and Gynaecology, Onze Lieve Vrouwe Gasthuis Amsterdam, the Netherlands; 2 Department of Obstetrics and Gynaecology, Leiden University Medical Center, the Netherlands; 3 Department of Obstetrics and Gynaecology, University Medical Centre, Utrecht, the Netherlands; 4 Department of Obstetrics and Gynaecology, AMPHIA hospital, Breda, the Netherlands; 5 Department of Obstetrics and Gynaecology, Academic Medical Centre Amsterdam, the Netherlands; 6 Department of Obstetrics and Gynaecology, Academic Hospital Maastricht, the Netherlands; 7 Department of Obstetrics and Gynaecology, Erasmus Medical Center Rotterdam, the Netherlands; 8 Department of Obstetrics and Gynaecology, University Medical Center St Radboud Nijmegen, the Netherlands; 9 Department of Obstetrics and Gynaecology, Máxima Medical Center Veldhoven, the Netherlands; 10 Department of Obstetrics and Gynaecology, Isala Hospital, Zwolle, the Netherlands; 11 Department of Obstetrics and Gynaecology, VU Medical Center Amsterdam, the Netherlands; 12 Department of Obstetrics and Gynaecology, University Medical Center Groningen, the Netherlands

## Abstract

**Background:**

Multiple pregnancies are at high risk for preterm birth, and therefore an important cause of infant mortality and morbidity. A pessary is a simple and potentially effective measure for the prevention of preterm birth. Small studies have indicated its effectiveness, but large studies with sufficient power on the subject are lacking. Despite this lack of evidence, the treatment is at present applied by some gynaecologists in The Netherlands.

**Methods/Design:**

We aim to investigate the hypothesis that prophylactic use of a cervical pessary will be effective in the prevention of preterm delivery and the neonatal mortality and morbidity resulting from preterm delivery in multiple pregnancy. We will evaluate the costs and effects of this intervention. At study entry, cervical length will be measured. Eligible women will be randomly allocated to receive either a cervical pessary or no intervention. The cervical pessary will be placed in situ at 16 to 20 weeks, and will stay in situ up to 36 weeks gestation or until delivery, whatever comes first.

The primary outcome is composite bad neonatal condition (perinatal death or severe morbidity). Secondary outcome measures are time to delivery, preterm birth rate before 32 and 37 weeks, days of admission in neonatal intensive care unit, maternal morbidity, maternal admission days for preterm labour and costs. We need to include 660 women to indicate a reduction in bad neonatal outcome from 7.2% without to 3.9% with a cervical pessary, using a two-sided test with an alpha of 0.05 and a power of 0.80.

**Discussion:**

This trial will provide evidence on whether a cervical pessary will decrease the incidence of early preterm birth and its concomitant bad neonatal outcome in multiple pregnancies.

**Trial registration:**

Current Controlled Trials: NTR 1858

## Background

Twin pregnancies are at high risk for preterm birth. In The Netherlands 15% of the women with a multiple pregnancy deliver before 34 weeks of gestation[1]. About 1 in 60 pregnancies is a twin pregnancy, and about 30% of the preterm born children admitted in a neonatal care (NICU) are from twin pregnancies. [2,3]. The incidence of twin pregnancies remains high due to an increase in the age of pregnant women. Prevention of preterm birth is therefore a major goal of obstetric care. However, strategies to prevent preterm birth have been largely unsuccessful.

Bad neonatal outcome includes respiratory distress syndrome (RDS), bronchopulmonary dysplasia (BPD), intraventricular haemorrhage (IVH), periventricular leucomalacia (PVL), necrotizing enterocolitis (NEC), sepsis and death before discharge [4]. The prevalence of this poor neonatal outcome is 77%, 35% and 12% in children born after early preterm delivery between 24-27, 28-32 and 32-34 weeks, respectively [3]. But after 34 weeks this incidence sharply declines to less than 2% at term. The probability that a woman with a multiple pregnancy delivers at these gestational ages is 1.8%, 5.4% and 7.2%, respectively [2].

In total, about 8% of the multiple pregnancies will result in the death of at least one child, whereas in 7% of the pregnancies at least one of the children will remain severely disabled. Moreover, another 20% of the pregnancies results in a moderate handicap of at least one of the children.

The cost of these poor outcome is enormous. About 500 of these children will be admitted into a neonatal intensive care unit at a cost of € 1500,- per day, with an average duration of admission of 10 days. This results in a total cost of 16 million euro per year from the birth of 1.000 children before 34 weeks.

A pessary is a simple and potentially effective measure for the prevention of preterm birth.

Preliminary evidence on the effectiveness of a pessary to prevent preterm labour is promising. Various designs of pessary have been used[5] but none has been evaluated in a well-designed randomised trial. In the only randomised controlled trial of pessary prophylaxis comparing pessary to cerclage [6], the type of pessary used and the study methodology were not well described. A double ring shaped pessary is at present the most popular pessary, and widely used in The Netherlands and Germany with the manufacturers selling around 2000 per year.

The first reports show a strong beneficial effect of a pessary in women with singleton pregnancies and a short cervix [7]. A cohort study in twins showed that a pessary prolonged pregnancy and decreased delivery before 32 weeks [8]. A prospective cohort study by Acharya et al. also used an double ring shaped pessary (Arabin-pessary) to treat cervical incompetence in women with a cervical length ≤ 25 mm, before 30 weeks [9]. This study showed that in 55 percent of the patients, the pregnancy could be postponed to 34 weeks or more. In view of these data, we will set up a randomised clinical trial on the subject. The aim of this trial is to investigate whether a prophylactic cervical pessary will lower the incidence of neonatal morbidity by reducing the number of preterm births in multiple pregnancy.

## Methods/Design

### Aims

We will use a randomised trial (the ProTwin-trial: Pessaries in multiple pregnancy as a prevention of preterm birth: the ProTwin Trial) to assess the effect of a cervical pessary on neonatal outcome.

The study is set in the Dutch Obstetric Research Consortium, a collaboration of obstetric practices in the Netherlands. Approximately 50 clinics, including academic hospitals, non-academic teaching hospitals and non-teaching hospitals will participate in this trial.

To conduct this trial we got approval from the Medical Ethics Committee, Academic Medical Centre, Amsterdam, The Netherlands (MEC O9/1O7).

### Participants

All women presenting with a multiple pregnancy between 12 and 19 weeks of gestation are eligible for the study. We propose to also include women with monochorionic pregnancies as well as women with a triplet pregnancy or women with a previous preterm birth. Women with multiple pregnancies in which at least one of the foetuses has major congenital anomalies known at study entry will not be included. Gestational age will be determined from the menstrual history and confirmed from the measurement of foetal crown-rump length (of the bigger twin) at a first-trimester scan.

### Procedures, recruitment, randomisation and collection of baseline data

All eligible women who present at one of the participating clinics will be referred to an obstetrician or a specifically appointed research nurse/midwife for counselling. They will receive an information sheet and, where possible, are given two weeks time to reflect on participation.

Once a patient has given informed consent, and the patient data have been entered in a web-based database, randomisation will be done over the internet.

Randomisation will be stratified for centre, parity (previous vaginal delivery or not), chorionicity (multichorial versus monochorial) and number of multiples (twin or higher order gestation).

We will apply block randomisation with a variable block size and a minimalisation procedure. The pessaries will be packaged according to the randomisation sequence at the central consortium office in Amsterdam, the Netherlands. Randomisation will be 1:1 for cervical pessary versus no treatment.

The study will not be blinded.

Baseline demographic, past obstetric and medical histories will be recorded for all women. Cervical length will be measured by transvaginal ultrasound at the time of randomisation or at the next visit.

### Intervention

Eligible women will be randomly allocated to receive either a cervical pessary (Arabin pessary) or no intervention. The cervical pessary will be placed in situ at 16 to 20 weeks, and will stay in situ up to 36 weeks gestation or until delivery, whatever comes first.

All women participating will have a transvaginal measurement for cervical length between a gestational age of 18 to 22 weeks. Next to this research intervention cases are treated according to the local protocol in the participating clinics and other interventions i.e. tocolysis and corticosteroids in case of a threatened preterm birth can be carried out as usual. Furthermore no extra interventions will be needed. The use of progesterone is allowed, but this will mainly depend on the outcome of our current AMPHIA study[10].

### Outcome measures

The main outcome parameter is the composite morbidity rate of children in the two groups. This composite morbidity rate contains the following variables: periventricular leukomalacia (PVL), severe Respiratory Distress Syndrome (RDS), Broncho Pulmonal Dysplasia (BPD), Intraventricular Haemorrhage II B or worse, Necrotizing Enterocolitis (NEC), proven sepsis and death before discharge from the nursery 24. They will be measured until 6 weeks after the expected term date of delivery.

Secondary outcome measures are time to delivery, preterm birth rate before 32 and 37 weeks, days of admission in neonatal intensive care unit, maternal morbidity, maternal admission days for preterm labour and costs. At present, a longer follow-up is not planned.

### Follow up of women and infants

All details of delivery, maternal assessments and admittance during pregnancy are recorded in the case record form that is accessible through the website. In case of admittance of one or more children to the neonatal intensive care unit, details of this admittance are also recorded. The main outcome parameter, a composite neonatal mortality and morbidity, will be measured until 6 weeks after the expected term date of delivery.

Long-term follow up of children is desirable, but is depending on future funding

### Economic evaluation

#### General considerations

The economic evaluation will be performed from a societal perspective, with the costs per unit reduction in the primary outcome ('bad neonatal outcomes') as the primary outcome measure. The appropriate type of economic evaluation is conditional on the results. We hypothesize that when a pessary is effective, this intervention will be associated with less neonatal health care utilization, as well as less burden to parents (time costs) and absence from paid work (productivity costs). Therefore, the primary analysis will be a cost-effectiveness analysis that evaluates costs associated with an improved neonatal outcome, expecting the peccary intervention to be dominant (better health at lower costs). With a study horizon of 12 months after delivery, no discounting will be applied. Study specific costs will be excluded from the analyses.

### Cost analysis

We will differentiate between direct medical, direct non-medical and indirect costs. Within direct medical costs, the process of care is distinguished into three stages: antenatal stage, delivery/childbirth and postnatal stage. In the antenatal stage, costs are generated by health care utilization related to the placement and check of the peccary, hospital care and outpatient visits. Costs during childbirth are dominated by the course of childbirth and type of delivery. In the postnatal stage, costs are generated by hospital-based maternal care (hospitalisation etc.), neonatal care (admission to NICU/medium care or maternity ward, related diagnostic care and treatment, outpatient visits), primary care and informal care.

If neonatal health is suboptimal, further direct medical, direct non-medical and indirect costs may occur. Hence, for these infants, resource use of infants and/or parents is measured during 12 months after childbirth. Direct non-medical costs in the antenatal and postnatal stages are generated by travel to and from health care providers, and time costs as the result of household shifts. Indirect costs are associated with lost productivity due to absence from paid work, predominantly in the postnatal stage.

### Statistical issues

#### Sample size

The sample size is calculated based on the primary outcome 'bad neonatal outcome'. In the control group, 'bad neonatal outcome' is expected in 7.2% of the children (1.8%*77% + 5.4%*35% + 7.2%*12% + 35.6%*8% + 50%*.5% = 7.2%). In this calculation, the first rate represents the probability that a patient delivers at that gestational age, whereas the second rate represents the probability of 'bad neonatal outcome' at that particular gestational age. In case of treatment, 'bad neonatal outcome' is then expected in 3.9% of the children (0.9%*77% + 2.7%*35% + 3.6%*12% + 17.8%*8% + 75%*.5% = 3.9%).

Because the outcomes in children from multiple pregnancies are to a certain extent non-independent, we adjusted our sample size assuming a correlation of 0.6 for composite neonatal morbidity between two children born from the same pregnancy[11]. Using a two-sided test with an alpha of 0.05 and a power of 0.80 we need 330 women in the control group and 330 in the intervention group (total of 660 women).

### Data analysis

Data will initially be analysed according the intention to treat method. The main outcome variable, 'bad neonatal outcome', will be assessed by calculating rates in the two groups, relative risks and 95% confidence intervals as well as numbers needed to treat. To evaluate the potential of each of the strategies, we will also perform a par protocol analysis, taking into account only those cases that were treated according to protocol.

Time to delivery will be evaluated by Kaplan-Meier estimates, with account for differing durations of gestation at entry, and will be tested with the logrank test. The other secondary outcome measures will be approached similarly to the primary outcome measure.

We plan a separate analysis of women with a cervical length below 25 mm at 18 tot 22 weeks, and we will look at interaction between cervical length and treatment effect.

### Interim analysis

An interim analysis will be performed after the inclusion of 300 women. This analysis will be done by an independent data and safety monitoring committee that will not be aware of the allocation of pessary or no treatment when they judge data on effectiveness. In case of severe side-effects, the safety monitoring committee can advise to stop the study.

## Discussion

In the past, a lot of strategies have been used to prevent preterm birth in singleton and multiple pregnancies, like bed rest, uterine activity monitoring, prophylactic tocolysis and primary cerclage. However, none of these strategies have been proven to be effective.

A pessary is a simple and potentially effective measure for the prevention of preterm birth. Small studies have indicated its effectiveness, but large studies with sufficient power on the subject are lacking. Despite this lack of evidence, the treatment is at present applied by some gynaecologists in The Netherlands. From that perspective, and taking into account the major medical and social implications of preterm birth, research has to be done into the possible beneficial effects of progesterone in multiple pregnancies

Simultaneously with the ProTwin-trial, a number of other study groups in different countries have set up trials to investigate the effectiveness of a cervical pessary in preventing preterm birth in singleton as well as in multiple pregnancies. There is a trial in Spain [7], one in France [12] and one in the United Kingdom [13]. This last study will include patients in Brazil, Chile, Colombia, Germany, India, Portugal, Spain and the United Kingdom. If the outcome data of these studies are pooled, a more conclusive statement can be made on this matter.

## Abbreviations

NICU: Neonatal Intensive Care Unit; AMPHIA: 17-**A**lpha hydroxyprogesterone in **M**ultiple pregnancies to **P**revent **H**andicapped **I**nf**A**nts;

## Competing interests

The authors declare that they have no competing interests.

## Authors' contributions

BWM, DJB and HWB were involved in conception and design of the study. MAH, DJB and BWM drafted the manuscript. All authors mentioned in the manuscript are members of the ProTWIN study group. They participated in the design of the study during several meetings and are local investigators in the participating centres. All authors mentioned in the manuscript are members of the ProTwin study group. They are local investigators in the participating centres. All authors read and approved the final manuscript.

## Pre-publication history

The pre-publication history for this paper can be accessed here:


