# Correction: Pessaries in multiple pregnancy as a prevention of preterm birth: the ProTwin Trial

**DOI:** 10.1186/1471-2393-12-37

**Published:** 2012-05-23

**Authors:** Sophie MS Liem, Dick J Bekedam, Kitty WM Bloemenkamp, Anneke Kwee, Dimitri NM Papatsonis, Joris AM van der Post, Arianne C Lim, Hubertina CJ Scheepers, Christine Willekes, Johannes J Duvekot, Marc Spaanderman, Martina Porath, Jim van Eyck, Monique C Haak, Marielle G van Pampus, Hein W Bruinse, Ben Willem J Mol, Maud A Hegeman

**Affiliations:** 1 Department of Obstetrics and Gynaecology, Onze Lieve Vrouwe Gasthuis Amsterdam, Amsterdam, the Netherlands; 2 Department of Obstetrics and Gynaecology, Leiden University Medical Center, Leiden, the Netherlands; 3 Department of Obstetrics and Gynaecology, University Medical Centre, Utrecht, the Netherlands; 4 Department of Obstetrics and Gynaecology, AMPHIA hospital, Breda, the Netherlands; 5 Department of Obstetrics and Gynaecology, Academic Medical Centre Amsterdam, Amsterdam, the Netherlands; 6 Department of Obstetrics and Gynaecology, Academic Hospital Maastricht, Maastricht, the Netherlands; 7 Department of Obstetrics and Gynaecology, Erasmus Medical Center Rotterdam, Rotterdam, the Netherlands; 8 Department of Obstetrics and Gynaecology, University Medical Center St Radboud Nijmegen, Nijmegen, the Netherlands; 9 Department of Obstetrics and Gynaecology, Máxima Medical Center Veldhoven, Veldhoven, the Netherlands; 10 Department of Obstetrics and Gynaecology, Isala Hospital, Zwolle, the Netherlands; 11 Department of Obstetrics and Gynaecology, VU Medical Center Amsterdam, Amsterdam, the Netherlands; 12 Department of Obstetrics and Gynaecology, University Medical Center Groningen, Groningen, the Netherlands

## 

The initial sample size calculation in our protocol [1] was based on the expected proportion of ‘bad neonatal outcome’ in the intervention group (3.9%) and control group (7.2%) and accounts for the fact that the outcomes in children from multiple pregnancies are non-independent using an intra class correlation of 0.6. As the intervention is performed on the mother, analysis should be done on the maternal level. This adjustment was made during recruitment and approved by the medical ethics committee. The sample size is calculated based on the primary outcome 'bad neonatal outcome'. In the control group, 'bad neonatal outcome' is expected in 7.2% of the children $\left(1.8\%*77\%+5.4\%*35\%+7.2\%*12\%+35.6\%*8\%+50\%*.5\%=7.2\%\right)$. In this calculation, the first rate represents the probability that a patient delivers at that gestational age, whereas the second rate represents the probability of 'bad neonatal outcome' at that particular gestational age. In case of treatment, 'bad neonatal outcome' is then expected in 3.9% of the children $\left(0.9\%*77\%+2.7\%*35\%+3.6\%*12\%+17.8\%*8\%+75\%*.5\%=3.9\%\right)$. On the mother level this corresponds to an expected ‘bad neonatal outcome’ in at least one of two children of 12.4% in the control group and 6.7% in case of treatment. Using a two-sided test with an alpha of 0.05 and a power of 0.80 we need 400 women in the control group and 400 in the intervention group.

* Medical Ethics Committee, Academic Medical Centre, Amsterdam, the Netherlands (ref. No. MEC 09/107).

## Pre-publication history

The pre-publication history for this paper can be accessed here:

http://www.biomedcentral.com/1471-2393/12/37/prepub
